# Collectanea Medica

**Published:** 1814-06

**Authors:** 


### COLLECTANEA MEDICA,

CONSISTING OF

### ANECDOTES, FACTS, EXTRACTS, ILLUSTRATIONS, QUERIES, SUGGESTIONS, &c.

RELATING TO THE

*History or the Art of Medicine, and the Auxiliary Sciences.*

### *State of Medical Practice and Diseases in Tripoli, in the year* 1811; *by* E. Blaquiere, Esq.

THE number of physicians throughout the regency is not equal to what we have in the London Hospital; and such is the uninterrupted health enjoyed by all classes of the community, that these have but little practice. Their fees seldom exceed sixpence; even operations are performed for a shilling! Nothing is internally administered to patients except herbs of different kinds; regimen is the great resource during sickness. The surgical instruments would doubtless excite the curiosity of our faculty; they consist of a few irons of different sizes, with figures marked on the ends: these are applied to various parts of the body, as the nature of diseases requires; their miraculous effect, in many instances that have come within my own observation, furnishes a most interesting ground of speculative inquiry as to the effects of fire and heat on the human system. However incredible the assertion may appear, I have, during my intercourse with Tunis and this place, known several attacks of rheumatism to be removed by the application of these irons, and one case, in which a person suffering from the effects of a rupture received the greatest relief. The instrument is usually applied behind one of the ears, and often under the ancles: wounds are generally burnt out by them, and ulcers of a serious nature are frequently cured.

There are no public hospitals; and cripples, or people of a deformed appearance, are never seen in public.

Broken bones and fractures are very successfully treated in general; the ostrich grease is universally applied to them and contusions of every description. Amputations are performed with amazing celerity; the arteries are not taken up as in Europe; and a limb is no sooner taken off, than the stump is dipped into a bowl of hot pitch, which generally stops the bleeding, and soon brings about a cure. In fact, when we reflect on the simplicity of treating diseases in this almost savage country compared with the innumerable medical impositions peculiar to our own, it is impossible not to deplore the progress of refinement, which, if it has raised us above other people in so many ways, has also created a number of imaginary evils unknown to a less civilized state of society.—*Letters from the Mediterranean*, 1813.

Quarterly

*Quarterly Report of the Diseases occurring in Boys at Christ's Hospital, with a Synoptical Table, and Practical Observations; by* HENRY FIELD, Member of the Society of Apothecaries, London, and Apothecary to Christ's Hospital.

Christ's Hospital, although placed nearly in the centre of London, may be considered as occupying a very healthy situation. It stands adjoining to the highest spot of ground in the city, and comprises, within its boundaries, an area of several acres of land, a considerable part of which is allotted as a place for the play and recreation of the boys, which renders the buildings sufficiently open, and thus admits a free circulation of air. Several of the wards or dormitories are well adapted for the purposes to which they are applied, while others of them are not so lofty as might be wished. This arises from the buildings not having been originally erected for their present uses, but accommodated to them, from time to time, as necessity required, or circumstances would allow.

The hall, or eating-room, is spacious and convenient; as are likewise the several school-rooms.

The boys are not admitted under the age of seven years, nor permitted to remain in the school longer than that of fifteen; with the exception of a few of them, who are intended either for the university, or for the sea service. As much the greater part of the younger boys, at their first admission, are sent to the school at Hertford, where they continue three or four years, and are then brought up to London; a very large proportion of those in London may be considered as being between the ages of eleven and fifteen years.

Their diet is plain and simple; the allowance of bread is very ample and of the best quality; that of animal food is in quantity moderate, and of culinary vegetables but small: but the great quantity of bread allotted to them, renders the latter less necessary, and thus, without material inconvenience, they are dispensed with.

Their clothing is sufficiently warm. They generally go about in the open air with their heads uncovered, the small round caps, which are part of their costume, not being well adapted to that purpose; but it does not appear that this is attended with effects prejudicial to their health. Perhaps the circumstance of being almost constantly uncovered, may render them less susceptible of the impressions of cold, than if they were sometimes covered and sometimes exposed; which is generally the case with boys of the same age at other schools, whose hats, even when out of the house, are as often off their heads as on them, particularly during the hours of play.

Some of the boys educated in Christ's Hospital have been delicately brought up, being the children of parents once in the higher or more opulent stations of life, but reduced in their circumstances; others, on the contrary, are derived from the lowest stations, and from necessitous parents: but much the largest proportion are the children of clergymen, and of persons in the middle walks of life. The general constitutions of the boys may, therefore, be expected to be healthy and vigorous; neither, on the one hand, much enervated by excess of care and indulgence in infancy, nor, on the other hand, debilitated and impoverished by neglect, too great exposure to inclement seasons with inadequate clothing, or a diet, defective in quantity, and perhaps ill calculated for the purposes of nutrition. Their state of health is, for the most part, found to be such as to justify these expectations.

These introductory remarks might be much extended, but they will probably be esteemed sufficient to afford the previous knowledge that is necessary to our immediate subject.

It being my intention to commit to writing a quarterly report of the diseases occurring in the Infirmary of Christ's Hospital, it will be desirable to divide the year in such manner, as to correspond, as nearly as possible, to the four usual seasons. The winter season will therefore comprise the months of December, January, and February; the spring season, those of March, April, and May; the summer season, June, July, and August; the autumnal season, September, October, and November.

The diseases will be enumerated in the tabular form; to which will be subjoined such observations as shall be requisite to elucidate those cases which may be esteemed worthy of particular attention; or, to convey such general information as may appear calculated to promote the science and improve the practice of medicine.

| TABLE OF DISEASES. | 1813. December. | 1814. January. | 1814. February. |
|---|---|---|---|
| 1 Cynanche Tonsillaris | 9 | — | 1 |
| 2 Cynanche Trachealis | — | 2 | 1 |
| 3 Rheumatismus acutus | 2 | 1 | — |
| 4 Tussis Catarrhalis | 1 | 2 | 4 |
| 5 Febris Synochus | 8 | 3 | 4 |
| 6 Scarlatina Anginosa | 22 | 1 | 3 |
| 7 Enteritis | 1 | — | — |
| 8 Odontalgia Catarrhalis | 3 | 3 | — |
| 9 Obstipatio | 1 | 1 | 2 |
| 10 Otalgia | 1 | — | 1 |
| 11 Variola | — | 1 | 2 |
| 12 Peritonitis | — | 1 | — |
| 13 Pleurodyne | 3 | — | 1 |
| 14 Nausea, Gastrodynia vel Diarrhœa | 8 | 3 | 6 |
| 15 Urticaria | — | — | 4 |
| 16 Lichen simplex | — | — | 1 |
| Total | 59 | 18 | 30 |

*Observations.*—Notwithstanding the great severity of the weather during the month of January, it seems to have produced no ill effects upon the health of the boys in Christ's Hospital. The number of diseases occurring among them has been unusually small, when considered as one of the winter months. This is, however, no more than what might have been expected, it being consistent with general experience, that children at this period of life suffer but little inconvenience from the effects of cold, notwithstanding it may be extremely severe.

The

The only instance of fatality which has happened among us during the last three months, was in the case of Enteritis, reported in the month of December. Though the boy was only a few days in the Infirmary, yet there is reason to believe, from the appearances on dissection, that the disease must have been some considerable time coming on. The symptoms were by no means well defined, nor were there any very alarming appearances until about twenty-four hours before death, about which time mortification probably took place. The extent of mischief, which was afterwards discovered, was under these circumstances truly astonishing.

The body was opened about forty-eight hours after death, by Mr. Stanley, assistant to Mr. Abernethy, and a very skilful and accurate anatomist, who has favoured me with the following statement:

*Appearances on Dissection of D. P. aged 15 years.*—On opening the cavity of the abdomen, there were seen the effects of recent inflammation to the greatest extent, having pervaded every part of the peritoneum, both in the situation where lining the abdominal muscles, and in its reflections upon the viscera: an effusion of a turbid serous fluid, in quantity exceeding a pint, in which were floating flakes of coagulable lymph, had taken place into the cavity. The contiguous surfaces of the intestines were in many parts adherent. The jejunum, for an extent of two or three inches, appeared in a state of sphacelation. To this portion of the small intestine was connected a sac, apparently composed of an organised texture, and containing between three and four ounces of a dark-coloured fluid, having no particular odour. Its attachment to the peritoneal coat of the intestine was by a slender pedicle, but no continuity could be traced between the interior of the sac and the cavity of the intestine. The mucous lining of the stomach exhibited a healthy character, as well as that of the rest of the alimentary canal: the structure of the other viscera was natural.

The sac, or appendix, which was found adhering to the jejunum, is probably a very uncommon production, and whether congenial, or formed during life, it must certainly have been of very long standing.* As this sac discovered a higher degree of sphacelation than the other parts of the intestines, it is not improbable that the commencement of the disease might have taken place there, in the form of chronic inflammation, causing an exudation on the internal surface, gradually distending it to the size in which it appeared after death, and forming that peculiar fluid which it then contained.

Scarlatina Anginosa makes a prominent figure in this Table of Diseases, having been very prevalent among the boys during the whole of the present season. It commenced in the beginning of November, and has been, with a few exceptions, very mild in its form. The number of cases of this disease occurring in the Infirmary since that time were forty-eight, all of which terminated favorably. It has made its appearance at three different periods with-

---

* Something similar to this may be found in Morgagni de Sed. et Causis Morbor. Epist. 34, Art. 6.

in the last seven years. The whole number of boys, who have undergone the disease in that time, has exceeded one hundred and fifty; but not one instance of fatality under it has taken place. This may have arisen partly from the general mildness of the disease: but I have no doubt that considerable advantage is at all times enjoyed by the children of this house over those in private families, in the much earlier application of remedies to them when indisposed. In private families two, three, or more days often elapse, even when the morbid symptoms are severe, before medical advice is called for; during which time either nothing whatever is done, or only domestic and empirical remedies are employed. Though these remedies may have caused no positive injury to the patient, yet they must have been attended with the loss of invaluable time; during which the disease may not only have been acquiring strength, but the proper period for the application of powerful preventives may have passed by, and that stage of the malady arrived, in which such means cannot with safety be employed.

On the contrary, in Christ's Hospital, the nurses who have the charge of the boys when in health, have no concern in the management or care of them when ill, and are, therefore, under no temptation to neglect their duty in sending them to the Infirmary immediately upon the first appearance of indisposition.

Cynanche Trachealis is not an uncommon disease among the boys. Eleven cases of it have occurred during the year 1813. This is much beyond an average number, there having been only five instances of it in 1811, and two in 1812, exclusive of three cases which were connected with measles. Some of them were highly inflammatory, while in others the symptoms were mild, and the degree of inflammation moderate. The remarks, already made, upon the advantages attending the early application of suitable remedies, are in no cases more important than in the disease now before us; and the very great success, which has almost invariably followed the treatment of this dangerous malady, is too remarkable not to be considered as a striking confirmation of this opinion. I am perfectly aware that when this disease happens in early infancy, the speediest and most judicious application of remedies will often prove fruitless: this may be caused, partly, by the difficulty of carrying the evacuating plan to a sufficient extent at that age; and, partly, to the rapid vascular excitement which may then be expected to take place. I have long been of opinion that the prognosis of danger in Cynanche Trachealis may be generally formed, under equal degrees of disease, by the age of the patient: the probability of recovery increasing in a ratio nearly corresponding with the increase of years, at least until the age of puberty. How far this observation applies to adults, my experience is not sufficient to enable me to determine; but I have, within this month, had an instance of similar success in a youth about eighteen years of age.

The remedy, on which I would chiefly rely in this disease, is blood-letting, and that performed as expeditiously as possible. It has not been uncommon, in this house, for a boy of ten or twelve years years of age to have lost as many ounces of blood from the arm as early as within an hour or two after the first attack. The symptoms are almost always immediately alleviated; and though they will sometimes return after an interval of twelve or twenty-four hours, yet a repetition of bleeding, in quantity proportioned to the age and other circumstances of the patient, seldom fails to be attended with the best effects. At the same time, confinement to bed, a strictly antiphlogistic regimen, purgatives, especially with the sub-muriate of mercury, an emetic, antimonials in nauseating doses, and vesicatories, are some, or all of them to be employed, as the urgency of symptoms, or duration of the disease may require.

Tracheal inflammation is not uncommon during the febrile state, which exists prior to the eruption of measles. Under these circumstances a similar method of cure to that above described will generally prove successful, and the measles will follow without any unusual severity. I have seen three instances of Croup occurring on the decline of measles, or rather after the danger from that disease appeared to have entirely ceased. Every one of these cases proved fatal in less than forty-eight hours, notwithstanding the most vigorous and decisive application of remedies. They all happened about the same time in September 1807. The atmosphere was then so extremely cold for the season, that most families found it necessary to have recourse to fires, though only about the middle of the month; and to this cause I attributed this unusual event.

In the curative treatment of Croup, I omitted to mention topical bleeding by leeches. Though there are many cases in which they may be useful, and indeed necessary, particularly in very young subjects; yet, where the patient is of such an age as to admit the use of the lancet, it should always be preferred. In fact, early and copious bleeding is, in my opinion, indispensable, and by much the most important part of the curative plan.

Two late French writers on this disease, whose works I have not seen, but from which some extracts are inserted in the Appendix to the Monthly Review, vol. 72, do not appear to have insisted so much upon venesection as I could have wished; indeed, one of them seems to give the preference to topical bleeding by leeches. It is possible that the difference of climate and constitution, in a southern latitude, may render large evacuations of blood more improper than in our northern climate.

The instance of Variola mentioned in the January Report, was attended with circumstances worth recording.

G. M. Y. aged twelve years, was brought into the Infirmary on Thursday, Jan. 13th. He complained of pains in the head, and other parts of the body, with the usual symptoms of fever, of which he had a considerable degree: a slight convulsion fit followed. On the 16th an eruption appeared on the skin, soon discovering itself to be the small-pox, upon which the other symptoms abated. The eruption, though scarcely to be termed confluent, was as full as could well be without running together, and went on for three or four days in the usual manner, the pustules increasing, both as to

 dimension dimension and in quantity of fluid contents. After the fourth day, no further increase appeared to take place, the pustules gradually diminished, and on the ninth day, from the first eruption, were nearly all dried away. There were no alarming symptoms after the first two or three days, and the boy is now perfectly recovered.

Having understood from his parents that he underwent variolous inoculation in his infancy, I wrote to them, requesting information on the subject, and received, in answer, the following particulars from his father, a clergyman of the Church of England:—That, about eleven years ago, he was inoculated at the Small-pox Hospital, St. Pancras. He was then with his grandfather and grandmother at Camden Town, both of whom are since dead. The small-pox being in the neighbourhood, these relations, in the absence of his parents, considered it best for him to be inoculated; and on their return to town, they found their son well sprinkled with small-pox pustules.

The register books of that hospital have been carefully examined, for the space of three years, about the period alluded to, in order to obtain further satisfaction, but no trace of such inoculation can be discovered. The father is, nevertheless, perfectly certain of the fact, well recollecting that on his return to London, he met the person who had then the care of his child, coming out of the Hospital with him in her arms, and the eruption fully out upon him. It must, therefore, be supposed that he was registered there under a fictitious name; which is a circumstance I understand by no means uncommon, when the parties wish to remain unknown.

To remove all doubts of this secondary disease being truly variolous, I have to observe that the two cases of variola, contained in the February Table, were certainly received by infection from this boy.

Both the subjects of it had been previously vaccinated. The history of their vaccination I have had no opportunity of inquiring into; but from the appearances upon their arms, should suppose it to have been correctly performed. The secondary disease, in both instances, was very slight.

Having in my possession minutes of another case of small-pox after variolous inoculation, which has not been made public, I shall take the liberty of communicating it, though it occurred prior to the period of our present investigation.

G. I. was inoculated with the small-pox at Camden in Glocestershire when about two months old: the other children in the family were inoculated at the same time, as were also the servants; the children went through the disease in general very favourably, but some of the servants suffered much under it. The subject of our present notice is said not to have taken the disease on the first application of virus, and was therefore inoculated again, when he received the infection in so favourable a manner as to have had only two pustules. The appearance upon the arm at this time is such as to indicate much local action, having left a scar of considerable magnitude, closely surrounded with small marks or pits, apparently from those pustules,

 which which are well known frequently to encircle the place of insertion, when much inflammation is excited there, and is usually an indication that a mild disease will follow.

He was afterwards admitted into Christ's Hospital, and on the 28th of Sept. 1812, when about fourteen years of age, was attacked with symptoms of general fever. Three days afterwards a copious eruption appeared on the skin, which soon discovered itself to be the small-pox: the disease went through its usual stages with the most perfect safety, producing a very considerable crop of pustules, which maturated well, and on the twelfth day from the first appearance of eruption were nearly all exsiccated. I have reason to believe that he had not been, prior to this time, much exposed to variolous contagion, having resided chiefly in the country, either with his friends, or in the school at Hertford. The circumstances attending the inoculation were communicated by his relations.—*Apothecaries' Repository.*

---

*Case of an extensive Fracture of the Cranium, accompanied with Laceration of the Dura Mater and Cerebrum, that terminated favorably; by* WILLIAM SHEARLY, Surgeon, Deal.

Lieutenant Shepperd, Royal Marines, was wounded on-board his Majesty's ship Ariadne, by a musket ball, which struck him on the right parietal bone, close to the sagittal suture, whilst in action with a French flotilla off Boulogne.

On being landed at the Royal Naval Hospital, Deal, the 19th day of July 1805, the day after the accident, and at the time I was serving as senior assistant-surgeon to that establishment, the pupils of both eyes were much dilated, particularly the right, with coma and stertorous breathing. On examination, the wound of the scalp was found remarkably small; but on the application of the finger to the part, accompanied by moderate pressure, so extensive a fracture was discovered, as to render it necessary to expose the cranium from the right frontal tuberosity, to within half an inch below the lambdoidal suture. The part of the skull struck by the bullet was about the centre of the sagittal suture, where the dura mater was found lacerated to some extent, and the cerebrum wounded. A small quantity of brain was protruded through these lacerations, about the size of two peas, which afterward increased to that of a small hazel nut. So considerable was the fracture, that, on the first examination, twenty-two pieces of different portions of the frontal, parietal, and occipital bones, were removed, including both tables of the cranium, leaving the dura mater exposed to the extent of two inches and a half in a longitudinal direction, and one and a half transversely. All the detached portions of bone that were beaten down upon the dura mater, or thrust through that membrane, being removed, and the coagula of blood cleared away, the divided scalp was restored to its original position, as nearly as possible, by means of slips of sticking plaster; and the patient placed in bed.

Soon after, he evinced signs of returning animation; but his pulse for several hours was scarcely perceptible, owing most probably to the quantity of blood that issued from the divided arteries of the scalp during the operation. Dilatation of the pupils continued for two hours; but the contractile power being gradually restored, the patient sunk into a comfortable undisturbed sleep for several hours, from which he awoke much refreshed. As the patient's senses were gradually restored, all the different viscera resumed their customary functions, with the exception of the bowels, which remained obstinately torpid. It is scarcely necessary to add, that the treatment was strictly antiphlogistic.

The wounds had from the beginning a healthy appearance, without the least disposition to throw out fungus, either from the brain or the dura mater. During the cure, several small exfoliations took place of both tables of the skull. Mr. Shepherd's strength returned much sooner than might have been expected from this alarming and extensive injury done to parts so essential to life. The speech was much injured; and great irritability of disposition was manifested, as his recovery to perfect health advanced.

The foregoing case evidently shows the great regenerating powers of nature, and adds another proof to the few extant, that the dura mater and brain may be violently and extensively injured without fatal consequences ensuing. It proves also that the dura mater may be punctured with every chance of success, when effusion between that membrane and the brain is found to exist; which practice has been most fully exemplified by Dr. Hutchinson, surgeon to the Royal Naval Hospital at Deal, in a very valuable paper published by him, in the 2d volume of the Medico-Chirurgical Transactions. In Dr. H.'s case, it was the dura mater of the cerebellum that was punctured; the only instance of the kind on record. Mr. John Bell's remark, "that he had always found the puncture of the dura mater fatal," must therefore be considered merely as a caution to young practitioners.—*Apoth. Rep.*

---

*Experiments and Observations on the Influence of the Nerves of the eighth Pair on the Secretions of the Stomach; by* B. C. Brodie, Esq. F.R.S.—In a paper formerly communicated to the Royal Society by Sir Everard Home, and since published in the Philosophical Transactions for 1809, some facts were stated which render it probable that the various animal secretions are dependent on the influence of the nervous system; and this opinion seemed to derive support from some physiological experiments which were afterwards instituted by myself, and in which it was observed, that after the functions of the brain had been destroyed, although the action of the heart continued, and the circulation of the blood was maintained as under ordinary circumstances, the secreting organs invariably ceased to perform their office.

It has been attempted by former physiologists to determine how far the nerves are necessary to secretion, but there are considerable obstacles in the way of this inquiry, and no observations that have been hitherto made, appear to throw a great deal of light on the subject. The only method which can be devised of ascertaining, by direct experiment, whether the nerves are really necessary to secretion, tion, is that of dividing the nervous branches by which the glands are supplied; but this, with respect to the greater number of the glands, is an experiment impossible to perform, and, with respect to others, cannot be executed without so much disturbance and injury to the other parts, as must render it extremely difficult to arrive at any positive results. Perhaps, in future investigations, some circumstances may arise which will enable us to determine more satisfactorily this important physiological question. In the mean time, as the labours of physiologists have hitherto contributed so little to this purpose, any facts that tend to its elucidation may deserve to be recorded, and I am therefore induced to lay before the Society the following experiments, which afford one example of a secretion being dependent on the influence of the nerves.

The stomach derives its nerves principally from those of the eighth pair, or the *par vagum*; and the same nerves, as they assist in the formation of the semilunar ganglions, contribute to the supply of the rest of the alimentary canal, particularly of the small intestines. In an inquiry which I had formerly instituted, respecting the functions of the stomach, I divided these nerves in the neck of a dog, for the purpose of ascertaining the influence which they possess on the secretion of the gastric juice; but I was disappointed in my expectation, since the animals always died, in consequence of the disturbed state of the respiration, which the injury of the nerves occasioned, before there was an opportunity of ascertaining the effect produced on the process of digestion.

I had formerly ascertained, that in a dog poisoned by arsenic, there is a copious secretion of mucous and watery fluid from the mucous membrane of the stomach and intestines, which are in consequence found after death completely and preternaturally distended; and it occurred to me, that although I could not ascertain the effect of the division of the nerves of the eighth pair on the natural secretions of the stomach, it might be possible to ascertain the effect on a secretion thus artificially produced. With this view I instituted the following experiments.

*Exp.* 1.—The nerves of the eighth pair, with the accompanying sympathetic nerves, were divided in the neck of a dog, and immediately afterwards ten grains of arsenic were inserted into a wound of the thigh. The breathing became laborious, as is usual where these nerves are divided; and afterwards the same symptoms took place as commonly arise from the poison of arsenic, with this difference, that there was no discharge of fluid either from the stomach or intestines. He died at the end of three hours and a half. On dissection, the stomach and intestines were found to contain only food and fæces, there being none of the mucous and watery secretion usually met with in an animal which has been killed in the same manner. The mucous membrane of the stomach and intestines was highly inflamed.

*Exp.* 2.—The experiment was repeated on another dog. He died at the end of nine hours, and on dissection the stomach and intestines were not found to contain any mucous or watery fluid. Their mucous membrane was inflamed.

*Exp.*

*Exp.* 3.—A dog, immediately after the division of the same nerves in the neck, was made to swallow two ounces of saturated solution of white oxide of arsenic in water. He died at the expiration of three hours. On dissection, the stomach and intestines were found slightly inflamed, and they contained no mucous or watery fluid.

In these experiments, the animals died from the application of the arsenic, and the poison produced the usual symptoms, with the exception of the copious mucous secretion, which takes place in other instances from the stomach and intestines. The obvious conclusion was, that this secretion was prevented in consequence of the nervous influence having been interrupted by the division of the nerves of the eighth pair; but as this injury always induces a disturbed and laborious respiration, it was desirable to ascertain how far this circumstance might have operated towards the production of this effect, and I therefore repeated the experiment, but with this difference, that the nerves were divided in such a way as not to interfere with the functions of the lungs.

*Exp.* 4.—Having made an incision into the abdomen of a dog, immediately below the short ribs, I divided, by means of a bistoury, the stomachic ropes formed by the nerves of the eighth pair, where they are situated on the œsophagus, immediately above the cardiac orifice of the stomach. The wound was closed by sutures. The respiration was not in the least disturbed, but was performed as frequently, and with as much ease, as under ordinary circumstances. The animal was afterwards inoculated in the thigh with the white oxide of arsenic, and he died in a few hours after the application of the poison, with the ordinary symptoms, except that there were no fluid evacuations from the stomach or intestines.

On dissection, the mucous membrane of the stomach and intestines was found inflamed. There was no watery or mucous fluid in the stomach or small intestines. There was a small quantity of mucus in the colon.

The result of this being the same as that of the former experiments, we may conclude that the suppression of the secretions in all of them was to be attributed solely to the division of the nerves; and all the facts which have been stated sufficiently demonstrate, that the secretions of the stomach and intestines are very much under the controul of the nervous system. We cannot indeed venture to deduce from them any positive conclusions respecting the necessity of the nervous influence to the secretions in general, but as forming one link in the chain of an interesting but difficult physiological investigation, the circumstances which have been mentioned may perhaps be considered as possessing some value, and as worthy of being recorded.

It is proper to observe, that I have attempted to pursue the investigation, so as to ascertain the effect produced on the process of digestion by the division of the stomachic ropes on the termination of the œsophagus; but various circumstances, which it would be unnecessary to enumerate, have prevented my proceeding in the inquiry, and seem almost to render it impossible to make any observations on this subject.—*Phil. Trans.*

CRITICAL

